# Relationship between Patients' Baseline Characteristics and Survival Benefits in Immunotherapy-Treated Non-Small-Cell Lung Cancer: A Systematic Review and Meta-Analysis

**DOI:** 10.1155/2022/3601942

**Published:** 2022-05-19

**Authors:** Xuanbo Hu, Yafeng Liu, Yuxiao He, Zibo Wang, Hongyan Zhang, Wei Yang, Jibin Lu

**Affiliations:** Department of Thoracic Surgery, Shengjing Hospital of China Medical University, Shenyang, Liaoning, China

## Abstract

**Background:**

The difference of patients' baseline characteristics such as sex, age, Eastern Cooperative Oncology Group performance status (ECOG PS), and smoking status may influence the immune response. However, little is known about whether these factors affect the efficacy of immune checkpoint inhibitors (ICIs) in patients with advanced non-small-cell lung cancer (NSCLC). Therefore, we performed this systematic review and meta-analysis to investigate the relationship between patients' baseline characteristics and survival benefits in immunotherapy-treated NSCLC.

**Materials and Methods:**

We performed a systematic search of PubMed, the Cochrane Library, and Embase for randomized controlled trials (RCTs) of NSCLC immunotherapy. We also searched abstracts and presentations from the proceedings of the American Society of Clinical Oncology and the European Society of Medical Oncology to identify unpublished studies. These studies have available data based on patients' baseline characteristics (such as sex, age, ECOG PS, and smoking status). We take the hazard ratios (HRs) and 95% confidence intervals (CIs) of overall survival (OS) as the effect index and use the random effect model to pool the results.

**Results:**

We included 18 phase II/III RCTs with a total of 14,189 participants. The benefits of ICIs were found for both male (pooled OS-HR 0.77, 95% CI 0.72-0.82, *P* < 0.05) and female patients (pooled OS-HR 0.77, 95% CI 0.67-0.87, *P* < 0.05); for both younger (<65 y: pooled OS-HR 0.74, 95% CI 0.68-0.81, *P* < 0.05) and older patients (≥65 y: pooled OS-HR 0.80, 95% CI 0.75-0.86, *P* < 0.05); and for both patients with ECOG PS = 0 (pooled OS-HR 0.77, 95% CI 0.71-0.84, *P* < 0.05) and ECOG PS ≥ 1 (pooled OS-HR 0.76, 95% CI 0.70-0.82, *P* < 0.05). Moreover, there was no significant difference in the efficacy of ICIs among different sex (*P* value for interaction = 0.955), age (*P* value for interaction = 0.17), or ECOG PS (*P* value for interaction = 0.765). However, in patients with different smoking status, the application of ICIs significantly prolonged the OS of smokers (pooled OS-HR 0.77, 95% CI 0.71-0.83, *P* < 0.05) but could not significantly improve the OS of never smokers (pooled OS-HR 0.85, 95% CI 0.70-1.03, *P* > 0.05).

**Conclusions:**

ICIs could significantly improve prognosis in patients with advanced NSCLC, regardless of sex, age, or ECOG PS. But among patients with different smoking status, the survival benefits of never smokers treated with ICIs were no better than that of controls. The impact of these factors on immunotherapy should be considered in the future clinical practice and guidelines.

## 1. What Is Known and Objective

As we all know, lung cancer has long been one of the most common malignancies with the highest mortality rate all over the world [1, 2]. Clinically, since most patients have developed locally advanced or even distant metastasis at the time of diagnosis (57%), the 5-year relative survival rate of lung cancer is only 21% [2]. While platinum-based chemotherapy and radiotherapy remain the mainstay of advanced lung cancer treatment [3], the emergence of immune checkpoint inhibitors (ICIs) in the past decade is improving clinical outcomes for some patients with advanced cancer and changing the treatment landscape for non-small-cell lung cancer (NSCLC) [4, 5].

ICIs could enhance the body's antitumor immunity by restoring exhausted T cells in the tumor microenvironment, thus improving the durable response rate of some advanced cancer patients and providing a longer overall survival [6]. So far, because the favorable therapeutic effect of ICIs in some solid tumors and hematological tumors, several of them have been approved by the US Food and Drug Administration (FDA) for second-line or even first-line treatment of advanced NSCLC [7], including nivolumab (PD-1 inhibitor), pembrolizumab (PD-1 inhibitor), atezolizumab (PD-L1 inhibitor), durvalumab (PD-L1 inhibitor), avelumab (PD-L1 inhibitor), ipilimumab (CTLA-4 inhibitor), and tremelimumab (CTLA-4 inhibitor) [8–12]. However, due to the lack of reliable biomarkers to predict patient prognosis, further clinical application of ICIs remains a major challenge.

The immune system of the human body is affected by many external environment and self-factors. Patients may achieve different benefits from ICIs because of their different immune responses, but it is not clear which patients will benefit more from immunotherapy. It is known that sex and age are important variables affecting the human immune system. In general, both innate and acquired immunity are stronger in women than in men, leading to faster clearance of pathogens and a higher risk of autoimmune diseases in women [13]. As the body's immune system weakens with age, the risk of people dying from infection increases significantly, while the effectiveness of vaccinations decreases [14, 15]. Apart from these factors, some related studies have also confirmed that Eastern Cooperative Oncology Group performance status (ECOG PS) can significantly affect the immune response of human body [16]. Moreover, smoking status is not only highly related to the incidence of lung cancer but also has a significant impact on the efficacy and tolerance of many drugs for the treatment of lung cancer [17]. In terms of immunotherapy, smoking status also plays a role in the survival benefits of patients [18, 19]. Several previous meta-analyses have examined the effects of these factors on cancer immunotherapy [18–24], but few studies have specifically penetrated the field of lung cancer and have not reached consistent results.

Given that the influence of the above factors on the efficacy of immunotherapy remains hugely controversial, we performed this meta-analysis, incorporating the latest phase II/III clinical trials and establishing subgroup analyses to comprehensively research the relationship between advanced NSCLC patients' baseline characteristics with survival benefits of immunotherapy.

## 2. Materials and Methods

We performed the study in adherence with the Preferred Reporting Items for Systematic Reviews and Meta-analyses (PRISMA) guidelines [25], and the study protocol was registered with PROSPERO.

### 2.1. Search Strategy and Selection Criteria

First, we conducted a comprehensive literature search from three electronic databases (PubMed, the Cochrane Library, and Embase) to collect phase II/III randomized controlled trials (RCTs) of immunotherapy for advanced NSCLC from database establishment to June 1, 2021. Second, to identify unpublished studies, we searched abstracts and presentations from conference proceedings of the American Society of Clinical Oncology (ASCO) and the European Society for Medical Oncology (ESMO). The following terms were used for searching: non-small-cell lung neoplasm, cancer, or carcinoma; NSCLC; Immune Checkpoint Inhibitors; immunotherapy; PD-1; PD-L1; CTLA-4; nivolumab; pembrolizumab; atezolizumab; ipilimumab; avelumab; durvalumab; tremelimumab; ICIs; randomized controlled clinical trial; and RCT.

### 2.2. Selection Criteria

The following five criteria need to be met for inclusion: (1) RCTs of immunotherapy for advanced NSCLC; (2) data on the hazard ratio (HR) for death of overall survival (OS) reported based on participates' sex, age, ECOG PS, or smoking status must be available; (3) the intervention group received single ICI therapy or ICI combined with non-ICI therapy; (4) the control group should be treated without ICIs; and (5) phase II/III clinical trial published in English. If multiple reports of a study were available, we only included reports that contain the latest and most comprehensive data.

### 2.3. Data Extraction

Two authors independently extracted the data and resolved differences through discussions involving a third author until agreement was reached. We mainly extracted the following information from the studies: first author, journal name, number of participants, year of publication, trial name, pathologic type, treatment arms, and line of therapy. We also extracted the HR and 95% CI of the OS of the following predefined subgroups: sex (male vs. female), age (<65 y vs. ≥65 y), ECOG PS (0 vs. ≥1), and smoking status (never smokers vs. former/current smokers).

### 2.4. Statistical Analysis

Random-effects models were used for all meta-analyses because of the clinical heterogeneity inherent in the data. The HR and 95% CI of the OS were used as effect sizes. We used the *Q* test to determine the heterogeneity between studies and calculated the *I*^2^ values. The results were assessed using forest plots and presented as HRs for the main outcome. Egger's test and Begg's test were used to detect whether there is publication bias.

We further performed subgroup analyses to explore the impact of patients' different baseline characteristics on the survival benefits of immunotherapy. And the subgroups were line of therapy, intervention therapy, and pathologic types.

If the *P* value (two-sided) was less than 0.05, the results was considered to be statistically significant. All analyses were performed using STATA 16.0.

## 3. Results

### 3.1. Search Results and Patient Characteristics

**Figure 1:**
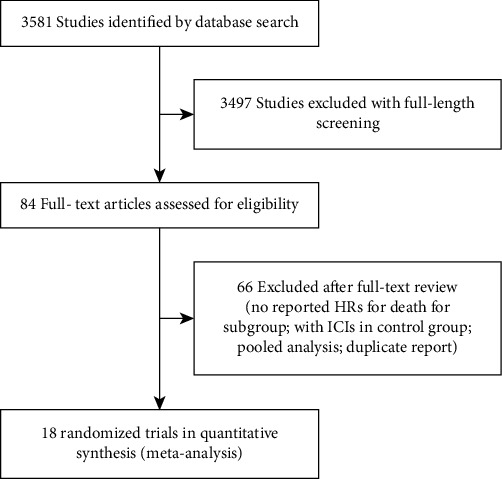
Flow diagram of literature search and selection process.

According to the research strategy, we retrieved a total of 3,581 articles, and after strict screening, a total of 14,189 participants from 18 studies met our criteria and were included in this meta-analysis [8–12, 26–38]. The specific selection procedure is shown in Figure 1.  Table 1 summarizes the detailed characteristics of each trial, of which 17 are phase III trials and one is phase II-III trials. 11 trials were for first-line treatment of advanced NSCLC and 7 trials for subsequence line therapy. 11 trials were treated with ICI alone, and 7 were treated with ICI combined with chemotherapy.

In addition, there is a study that needs further explanation. In the Impower150 trial [33], participants in the immunotherapy groups were randomly assigned to receive atezolizumab plus bevacizumab plus carboplatin plus paclitaxel (ABCP) and atezolizumab plus carboplatin plus paclitaxel (ACP), while the participants in the two control groups received the standard-of-care bevacizumab plus carboplatin plus paclitaxel treatment (BCP). Therefore, our meta-analysis divided this trial into ABCP vs. BCP group and ACP vs. BCP group for a pooled analysis.

### 3.2. Analysis according to Patients' Sex

**Figure 2:**
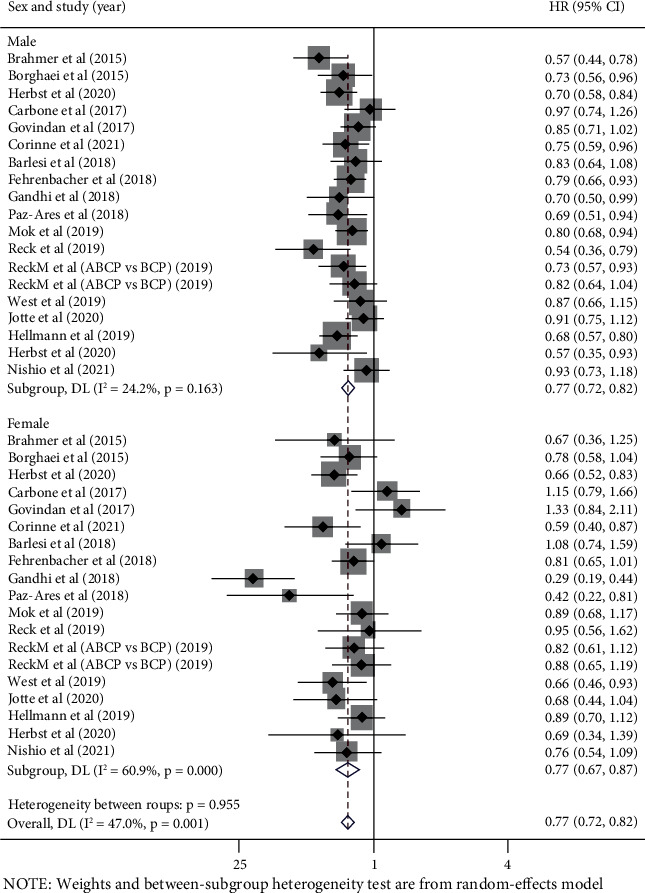
Forest plots of hazard ratios for overall survival of immunotherapy vs. control therapy in male and female.

A total of 13,311 participants in 18 trials reported OS-HR data based on patients' sex, 8,881 were male (66.7%), and 4,430 were female (33.3%). It was observed that the survival advantage of the immunotherapy was better than that of the control therapy in both male (pooled OS-HR 0.77, 95% CI 0.72-0.82, *P* < 0.05) and female patients (pooled OS-HR 0.77, 95% CI 0.67-0.87, *P* < 0.05), there was a significant interstudy statistically heterogeneity among female patients (*I*^2^ = 60.9%, *P* < 0.001), but not in male patients (*I*^2^ = 24.2%, *P* = 0.163) (Figure 2).

Compared to the control therapy, no significant difference was observed in the efficacy of immune checkpoint inhibitors between male and female (*P* value for interaction = 0.955). No significant difference was observed in subgroup analyses by different line of therapy, interventional therapy strategies, or pathological types (Table 2).

### 3.3. Analysis according to Patients' Age

**Figure 3:**
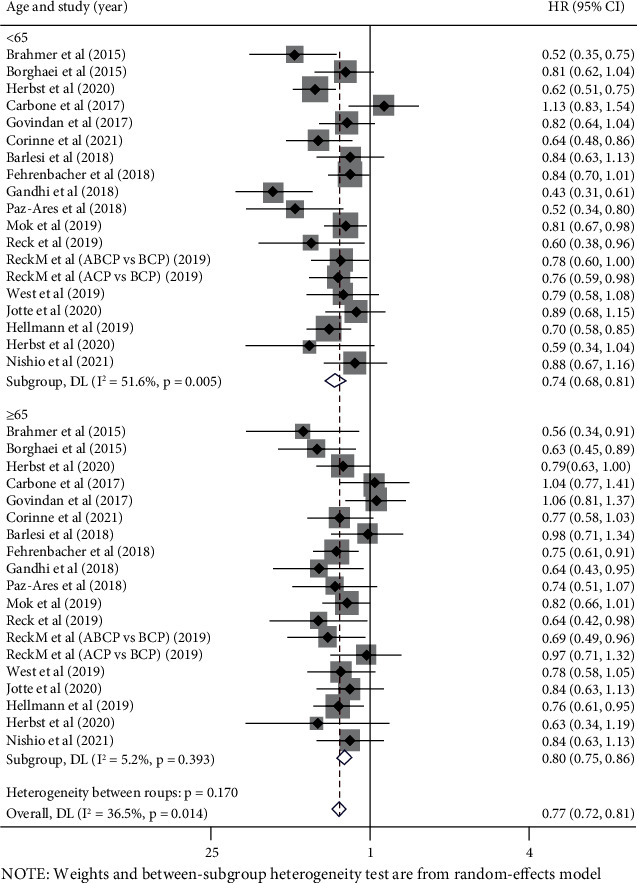
Forest plots of hazard ratios for overall survival of immunotherapy vs. control therapy in younger (age < 65) and older (age ≥ 65) patients.

A total of 12,807 participants in 18 trials reported OS-HR data based on patients' age, 7,092 were younger than 65 (55.4%), and 5,715 were 65 years or older (44.6%). The survival benefits of the immunotherapy were better than that of the control therapy in both younger (pooled OS-HR 0.74, 95% CI 0.68-0.81, *P* < 0.05) and older patients (pooled OS-HR 0.80, 95% CI 0.75-0.86, *P* < 0.05); there was a significant interstudy statistically heterogeneity among younger patients (*I*^2^ = 51.6%, *P* = 0.005), but not in older patients (*I*^2^ = 5.2%, *P* = 0.393) (Figure 3).

No significant difference was observed in the efficacy of immune checkpoint inhibitors between the two groups (*P* value for interaction = 0.17). And no significant difference was observed in subgroup analyses by different line of therapy, interventional therapy strategies, or pathological types (Table 3).

### 3.4. Analysis according to Patients' ECOG PS

**Figure 4:**
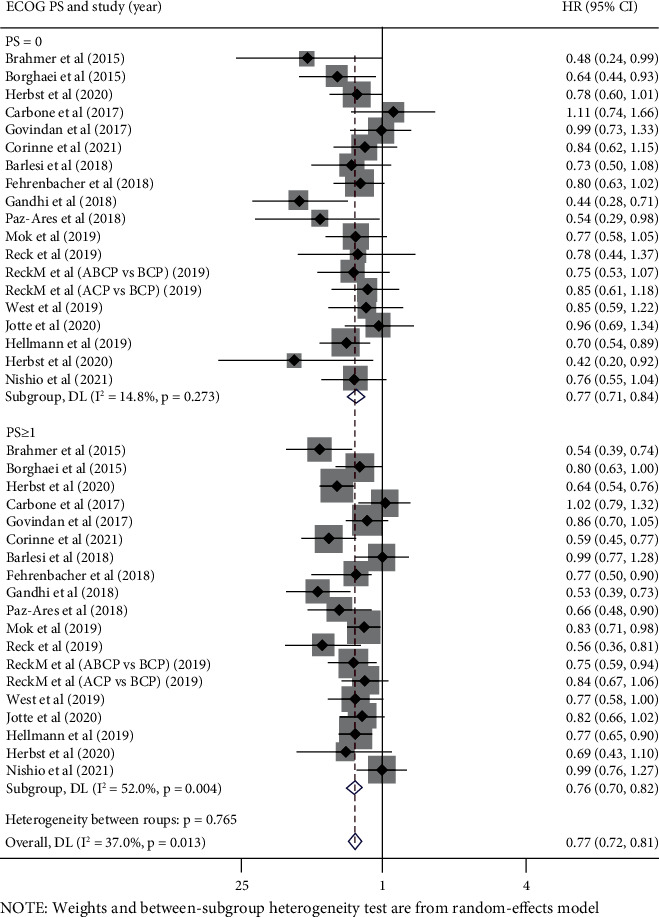
Forest plots of hazard ratios for overall survival of immunotherapy vs. control therapy in ECOG PS = 0 and ECOG PS ≥ 1 patients.

A total of 13,267 participants in 18 trials reported OS-HR data based on patients' ECOG PS, including 4,853 patients (36.6%) with ECOG PS = 0 and 8,414 patients (63.4%) with ECOG PS ≥ 1. The survival advantage of immunotherapy was superior to that of control therapy both in patients with ECOG PS = 0 (pooled OS-HR 0.77, 95% CI 0.71-0.84, *P* < 0.05) and ECOG PS ≥ 1 (pooled OS-HR 0.76, 95% CI 0.70-0.82, *P* < 0.05); interstudy statistically heterogeneity was not significant in either group (PS = 0: *I*^2^ = 14.8%, *P* = 0.273; PS ≥ 1: *I*^2^ = 37.0%, *P* = 0.013) (Figure 4).

Again, no significant difference was observed in the efficacy of immune checkpoint inhibitors between the two groups (*P* value for interaction = 0.765), and no significant difference was observed in subgroup analyses by different line of therapy, interventional therapy strategies, or pathological types (Table 4).

### 3.5. Analysis according to Patients' Smoking Status

**Figure 5:**
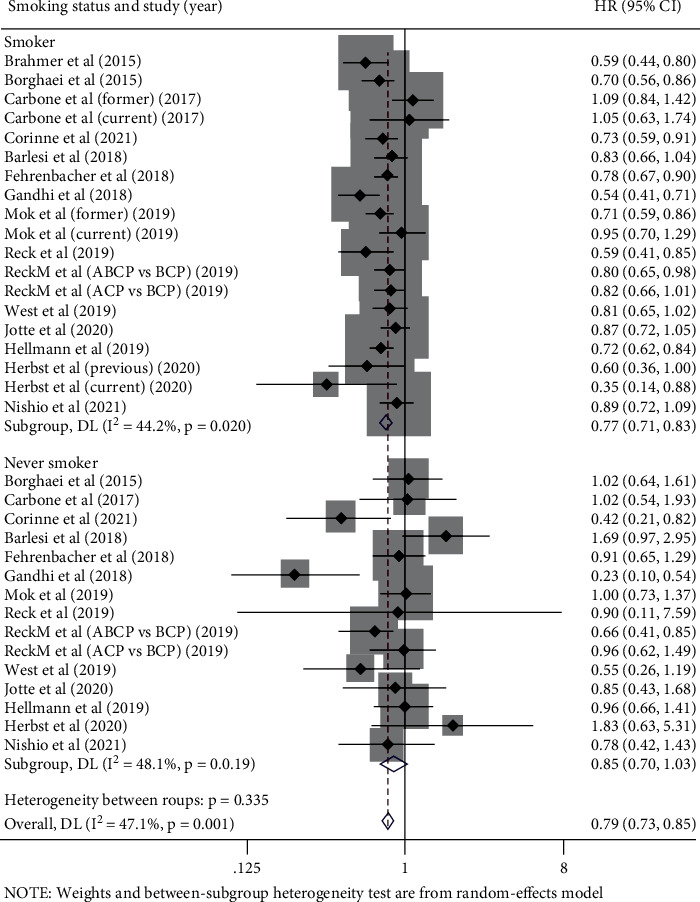
Forest plots of hazard ratios for overall survival of immunotherapy vs. control therapy in smokers and never smokers.

A total of 10,118 participants in 15 trials reported OS-HR data based on patients' smoking status, 8,679 were smokers (85.8%), and 1,439 were never smokers (14.2%). Our results suggested that immunotherapy significantly prolonged the OS in smokers (pooled OS-HR 0.77, 95% CI 0.71-0.83, *P* < 0.05) versus control therapy. However, among never smokers, no significant survival benefit was observed (pooled OS-HR 0.85, 95% CI 0.70-1.03, *P* > 0.05) compared with control therapy. Interstudy statistically heterogeneity was not significant in either group (smokers: *I*^2^ = 44.2%, *P* = 0.020; never smokes: *I*^2^ = 48.1%, *P* = 0.019) (Figure 5).

The same results were observed in subgroup analyses. Except in nonsquamous treatment settings and ICI combination therapy settings, the survival benefits of the immunotherapy group were superior to that of the control group in both smokers and nonsmokers. (Table 5).

### 3.6. Publication Bias and Sensitivity Analysis

**Figure 6:**
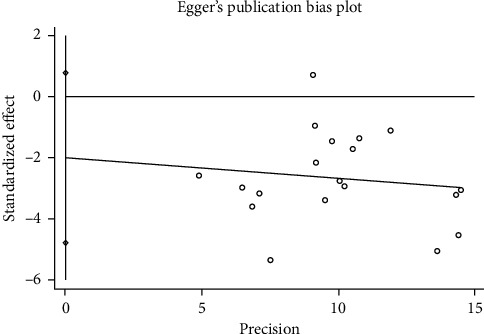
Egger's publication bias plot.

**Figure 7:**
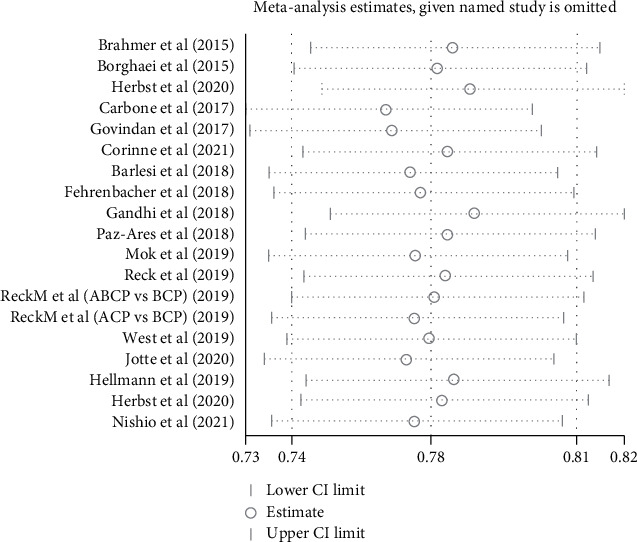
Sensitivity analysis.

Egger's test and Begg's test were used to verify whether there is a publication bias in our meta-analysis [39, 40], and the results showed that the publication bias is not significant (*P* = 0.148 for Egger's test; *P* = 0.184 for Begg's test) (Figure 6). Sensitivity analysis showed that there was no significant change in the comprehensive results after the deletion of any study (Figure 7).

## 4. Discussion

In the past decade, the rapid development of immunotherapy has brought a revolutionary breakthrough in the treatment of advanced NSCLC, showing a better effect than standard chemotherapy in certain patients. Currently, the primary contradiction is the lack of reliable biomarkers to identify which patients can benefit better from immunotherapy. Now, the most widely used makers are PD-L1 expression level, tumor mutation burden (TMB), and microsatellite instability (MSI) [41–43]. In addition, a series of trials have demonstrated that tumor infiltrating lymphocytes (TILs), exhaled breath analysis by use of eNose technology, and other biomarkers can help predict the efficacy of immunotherapy in patients with NSCLC [43–45]. However, these still need to be proved in more reliable experiments. In our study, we focused on the effects of sex, age, ECOG PS, and smoking status on immunotherapy, to investigate the relationship between these patients' baseline characteristics and survival benefits in immunotherapy-treated NSCLC.

In terms of sex, our results suggested that both men and women with advanced NSCLC benefit from immunotherapy, and no statistically significant differences were observed between the two groups. Furthermore, sex-related differences in efficacy were not observed when we performed subgroup analyses by different line of therapy, interventional therapy strategies, or pathological types. In a previous meta-analysis reported by Conforti et al. [46], the risk of death in male patients was statistically significantly reduced when treated with anti-PD-1/PD-L1 alone. In case of women, however, anti-PD-1/PD-L1 alone was not observed to be superior to standard chemotherapy. In contrast, female patients have better survival benefits than male patients in anti-PD-1/PD-L1 combined with chemotherapy. This is obviously quite different from the conclusion we have come to and the following reasons that may explain the above contradiction. First, the selection criteria of Conforti et al. were more stringent, and the OS analysis was based on only six first-line studies of NSCLC [12, 26, 30–32, 34], while OAK, CheckMate 017/057, PACIFIC, and other large trials were not included in their study. Second, our latest study included trials such as IMpower150, IMpower131, IMpower110, IMpower132, and CheckMate 227, which were conducted in the last two years since the results of Conforti et al. were published. These large trials contributed considerably to our pooled HR effect. To sum up, after our latest search results and a more specific evaluation of the research issues, as well as the inclusion of more immunotherapy agents, the current meta-analysis found no sex-related immunotherapy differences.

Several previous meta-analyses reported that younger (<65 y) and older (≥65 y) patients treated with ICIs had no significant difference in survival benefit [24, 47]. Our meta-analysis focused on advanced NSCLC, setting strict inclusion criteria, only including trials comparing ICI therapy with control therapy without ICI, and adding the latest phase III RCTs. We finally drew a similar conclusion; that is, no age-related differences in efficacy of immunotherapy were observed in patients with advanced NSCLC, and no statistically significant difference was observed in subgroup analyses. In addition, most of the trials we included took 65 years as the cut-off value, and only five of them divided the age into younger than 65, ≥65 to <75, and older than 75 [8, 27, 33, 35, 37]. Due to the lack of data on patients over 75 years old, we were concerned that we will not be able to get reliable results, so this group was not analyzed. As such, our results are not sufficient to reflect the true prognosis of participants over the age of 75 years. Therefore, in future studies, it is necessary to divide the age groups more carefully in order to explore the efficacy of ICIs in patients over 75 years old. For all that, no significant age-related difference in the efficacy of immune checkpoint inhibitors was observed according to the current analysis results, and we still support that immunotherapy should not be restricted by the age of patients in today's clinical treatment.

Recently, it has also been reported that ECOG PS may affect the immune response [16]. To our knowledge, there were no studies to confirm whether ECOG PS will affect the antitumor therapy of ICIs. Therefore, we also evaluated the heterogeneity of survival benefits of immunotherapy among different ECOG PS patients. Our results suggested that patients with advanced NSCLC with better or poorer ECOG PS could gain survival benefit from immunotherapy, no statistically significant difference was observed between the two groups, and there is no statistical difference in the results of subgroup analyses by line of therapy, interventional therapy strategies, or pathological types. It is worth noting that except for the PACIFIC trial and CheckMate 026 trial (the two trials classified patients' ECOG PS as equal to 0 and greater than or equal to 1), the other trials divide patients with different ECOG PS into 0 and 1 groups to represent patients with better and poorer conditions. Although no PS-related efficacy difference was observed in the results, due to the lack of relevant data in our included trials, caution should be exercised when treating patients with ECOG PS ≥ 2.

In the end, we also evaluated the relationship between patients' smoking status and survival benefits in immunotherapy-treated NSCLC. Although similar researches have been conducted in several previous meta-analyses [19, 22, 48], the results are open to debate due to the small number of trials included and the lack of detailed subgroup analysis. For these reasons, we thoroughly searched the database and included the latest high-quality trials. The results suggest that the using of ICIs significantly prolong survival in smokers with NSCLC compared with control groups, but not in never smokers. This is consistent with the result obtained by Li et al. [22] and El-Osta and Jafri [48]. Some studies have proved that there is a significant correlation between the clinical benefits of ICIs and TMB in patients with NSCLC. Smoking can significantly increase the TMB of patients, make the tumor more immunogenic, and thus increase the antitumor effect of ICIs [49]. However, our further subgroup analyses found that never smokers also gained survival benefits in the ICI combined therapy group and the nonsquamous NSCLC group. Therefore, we propose a hypothesis that in combined therapy group, chemotherapy may increase the efficacy of ICIs. However, there are no relevant studies to confirm this view, so further basic and clinical studies are still required. In conclusion, we believe that smoking status should be taken into full consideration when ICIs are used in the treatment of patients with NSCLC, and combination therapy may be more effective for never smoking patients.

## 5. What Is New and Conclusions

Our meta-analysis suggests that the survival benefits from immunotherapy in patients with advanced NSCLC with different sex, age (<65 y vs. ≥65 y), or ECOG PS (0 vs. ≥1) are similar to those in the control group, so they should not be limited by these factors when using ICIs. In patients with different smoking status, although ICIs can significantly improve the prognosis of smokers, for never smokers, ICIs have an advantage only in patients with nonsquamous NSCLC and patients treated with ICI combination therapy. The effect of this factor on immunotherapy of NSCLC patients should be taken into account in future clinical practice and guidelines.

## 6. Strengths and Limitations

As far as we know, our meta-analysis is the latest and most detailed assessment of the relationship between immunotherapy and various baseline characteristics in patients with advanced NSCLC. We included 18 phase II/III RCTs with a total of 14,189 participants. Supported by extensive clinical data, we comprehensively analyzed the effects of sex, age, ECOG PS, and smoking status on the survival benefits of immunotherapy for NSCLC and performed detailed subgroup analyses by patients' different line of therapy, intervention therapy, and pathologic types.

There are also some limitations in our results. First, because our analysis is based on published clinical trial data and lacks individual patient-level data, these factors hinder more in-depth analysis and may have potential publication bias. Second, there were few data reported by progress free survival (PFS) in the included trials, so we did not conduct further analysis based on PFS, which may require further discussion in future studies. In addition, since most of the studies did not include patients with ECOG PS ≥ 2 and older than 75 years old, our conclusions could not be well targeted at these two groups of people; more clinical data and further analysis are needed to improve this part in the future.

## Figures and Tables

**Table 1:** Characteristics of the studies and participants.

Source	Trial name	NCT #	Phase	Total pt #	Cancer type	Line of therapy	Treatment arms	Pt #	OS HR (95% CI)	Sex	Pt #	OS HR (95% CI)	Age	Pt #	OS HR (95% CI)	ECO G PS	Pt #	OS HR (95% CI)	Smoking status	Pt #	OS HR (95% CI)
2015-Brahmer-N Engl J Med	CheckMate 017	NCT01642004	3	272	Squamous	>1	Nivolumab vs. docetaxel	272	0.59 (0.44-0.78)	Male	208	0.57 (0.41-0.78)	<65	152	0.52 (0.35-0.75)	0	64	0.48 (0.24-0.99)	Current/former smoker	250	0.59 (0.44-0.80)
										Female	64	0.67 (0.36-1.25)	≥65 to <75	91	0.56 (0.34-0.91)	1	206	0.54 (0.39-0.74)			
2015-Borghaei-N Engl J Med	CheckMate 057	NCT01673867	3	582	Nonsquamous	>1	Nivolumab vs. docetaxel	582	0.75 (0.62-0.91)	Male	319	0.73 (0.56-0.96)	<65	339	0.81 (0.62-1.04)	0	179	0.64 (0.44-0.93)	Current/former smoker	458	0.70 (0.56-0.86)
										Female	263	0.78 (0.58-1.04)	≥65 to <75	200	0.63 (0.45-0.89)	1	402	0.80 (0.63-1.00)	Never smoked	118	1.02 (0.64-1.61)
2020- Herbst- J Clin Oncol	KEYNOTE-010	NCT01905657	2or3	1033	NSCLC	>1	Pembrolizumab vs. docetaxel	1033	0.69 (0.60-0.80)	Male	634	0.70 (0.58-0.84)	<65	604	0.62 (0.51-0.75)	0	347	0.78 (0.60-1.01)			
										Female	399	0.66 (0.52-0.83)	≥65	429	0.79 (0.63-1.00)	1	679	0.64 (0.54-0.76)			
2017- Carbone-N Engl J Med	CheckMate 026	NCT02041533	3	541	NSCLC	1	Nivolumab vs. chemotherapy	541	1.08 (0.87-1.34)	Male	332	0.97 (0.74-1.26)	<65	281	1.13 (0.83-1.54)	0	178	1.11 (0.74-1.66)	Former smoker	368	1.09 (0.84–1.42)
										Female	209	1.15 (0.79-1.66)	≥65	260	1.04 (0.77-1.41)	≥1	362	1.02 (0.79-1.32)	Current smoker	107	1.05 (0.63–1.74)
																			Never smoked	59	1.02 (0.54–1.93)
2017- Govindan-J Clin Oncol		NCT01285609	3	749	Squamous	>1	Ipilimumab+chemotherapy vs. placebo+chemotherapy	749	0.91 (0.77-1.07)	Male	635	0.85 (0.71-1.02)	<65	380	0.82 (0.64-1.04)	0	259	0.99 (0.73-1.33)	Heavy smoker	656	0.88 (0.73-1.05)
										Female	114	1.33 (0.84-2.11)	≥65 to <75	298	1.06 (0.81-1.37)	1	485	0.86 (0.70-1.05)	Former/light/nonsmoker	83	1.19 (0.71-1.99)
2021-Corinne- J Thorac Oncol	PACIFIC	NCT02125461	3	713	NSCLC	>1	Durvalumab vs. placebo	713	0.70 (0.57–0.86)	Male	500	0.75 (0.59–0.96)	<65	391	0.64 (0.48–0.86)	0	348	0.84 (0.62–1.15)	Smoker	649	0.73 (0.59–0.91)
										Female	213	0.59 (0.40–0.87)	≥65	322	0.77 (0.58–1.03)	≥1	365	0.59 (0.45–0.77)	Nonsmoker	64	0.42 (0.21–0.82)
2018-Barlesi- Lancet Oncol	JAVELIN lung 200	NCT02395172	3	529	NSCLC	>1	Avelumab vs. docetaxel	529	0.90 (0.73-1.12)	Male	367	0.83 (0.64-1.08)	<65	279	0.84 (0.63-1.13)	0	187	0.73 (0.50-1.08)	Ever smoker	444	0·83 (0·66–1·04)
										Female	162	1.08 (0.74-1.59)	≥65	250	0.98 (0.71-1.34)	1	342	0.99 (0.77-1.28)	Never smoker	84	1·69 (0·97–2·95)
2018- Fehrenbacher -J Thorac Oncol	OAK	NCT02008227	3	1225	NSCLC	>1	Atezolizumab vs. docetaxel	1225	0.80 (0.70-0.92)	Male	758	0.79 (0.66-0.93)	<65	661	0.84 (0.70-1.01)	0	455	0.80 (0.63-1.02)	Current/previous smoker	1017	0·78 (0·67–0·90)
										Female	467	0.81 (0.65-1.01)	≥65	564	0.75 (0.61-0.91)	1	770	0.77 (0.65-0.90)	Never smoked	208	0·91 (0·65–1·29)
2018-Gandhi- N Engl J Med	KEYNOTE- 189	NCT02578680	3	616	Nonsquamous	1	Pembrolizumab+chemotherapy vs. placebo+chemotherapy	616	0.49 (0.38-0.64)	Male	363	0.70 (0.50-0.99)	<65	312	0.43 (0.31-0.61)	0	266	0.44 (0.28-0.71)	Current/former smoker	543	0.54 (0.41–0.71)
										Female	253	0.29 (0.19-0.44)	≥65	304	0.64 (0.43-0.95)	1	346	0.53 (0.39-0.73)	Never smoked	73	0.23 (0.10–0.54)
2018-Paz- Ares-N Engl J Med	KEYNOTE- 407	NCT02775435	3	559	Squamous	1	Pembrolizumab+chemotherapy vs. placebo+chemotherapy	559	0.64 (0.49-0.85)	Male	455	0.69 (0.51-0.94)	<65	254	0.52 (0.34-0.80)	0	163	0.54 (0.29-0.98)			
										Female	104	0.42 (0.22-0.81)	≥65	305	0.74 (0.51-1.07)	1	396	0.66 (0.48-0.90)			
2019-Mok- Lancet	KEYNOTE- 042	NCT02220894	3	1274	NSCLC	1	Pembrolizumab vs. chemotherapy (PD-L1 TPS ≥ 1%)	1274	0.81 (0.71-0.93)	Male	902	0.80 (0.68-0.94)	<65	707	0.81 (0.67-0.98)	0	390	0.77 (0.58-1.05)	Never	282	1.00 (0.73-1.37)
										Female	372	0.89 (0.68-1.17)	≥65	567	0.82 (0.66-1.01)	1	884	0.83 (0.71-0.98)	Former	721	0.71 (0.59-0.86)
																			Current	271	0.95 (0.70-1.29)
2019-Reck-J Clin Oncol	KEYNOTE- 024	NCT02142738	3	305	NSCLC	1	Pembrolizumab vs. platinum-based chemotherapy	305	0.63 (0.47-0.86)	Male	187	0.54 (0.36-0.79)	<65	141	0.60 (0.38-0.96)	0	107	0.78 (0.44-1.37)	Current	65	0.81 (0.41-1.60)
										Female	118	0.95 (0.56-1.62)	≥65	164	0.64 (0.42-0.98)	1	197	0.56 (0.39-0.81)	Former	216	0.59 (0.41-0.85)
																			Never	24	0.90 (0.11-7.59)
2019-ReckM- Lancet Respir Med	IMpower150	NCT02366143	3	1202	Nonsquamous	1	ABCP vs. BCP	800	0.76 (0.63-0.93)	Male	479	0.73 (0.57-0.93)	<65	441	0.78 (0.60-1.00)	0	338	0.75 (0.53-1.07)	Never smoker	159	0.66 (0.41-0.85)
										Female	321	0.82 (0.61-1.12)	65-74	281	0.69 (0.49-0.96)	1	456	0.75 (0.59-0.94)	Current/previous smoker	641	0.80 (0.65-0.98)
							ACP vs. BCP	802	0.85 (0.71-1.03)	Male	480	0.82 (0.64-1.04)	<65	449	0.76 (0.59-0.98)	0	359	0.85 (0.61-1.18)	Never smoker	154	0.96 (0.62-1.49)
										Female	322	0.88 (0.65-1.19)	65-74	284	0.97 (0.71-1.32)	1	440	0.84 (0.67-1.06)	Current/previous smoker	648	0.82 (0.66-1.01)
2019-West- Lancet Oncol	IMpower130	NCT02367781	3	679	Nonsquamous	1	Atezolizumab+carboplatin+nab-paclitaxel vs. chemotherapy	679	0·79 (0·64–0·98)	Male	400	0.87 (0.66-1.15)	<65	341	0.79 (0.58-1.08)	0	280	0·85 (0·59–1·22)	Never smoker	65	0·55 (0·26–1·19)
										Female	279	0.66 (0.46-0.93)	≥65	338	0·78 (0·58–1·05)	1	397	0·77 (0·58–1·00)	Current/previous smoker	614	0·81 (0·65–1·02)
2020-Jotte-J Thorac Oncol	IMpower131	NCT02367794	3	1021	Squamous	1	Atezolizumab+carboplatin+nab-paclitaxel vs. carboplatin+nab-paclitaxel	683	0·88 (0·73–1·05)	Male	557	0·91 (0·75–1·12)	<65	326	0·89 (0·68–1·15)	0	225	0·96 (0·69–1·34)	Never smoker	55	0·85 (0·43–1·68)
										Female	126	0·68 (0·44–1·04)	65-74	279	0·84 (0·63–1·13)	1	456	0·82 (0·66–1·02)	Current/previous smoker	627	0·87 (0·72–1·05)
2019-Hellmann-N Engl J Med	CheckMate 227	NCT02477826	3	1739	NSCLC	1	Nivolumab+ipilimumab vs. chemotherapy	1166	0.73 (0.64–0.84)	Male	778	0.68 (0.57–0.80)	<65	611	0.70 (0.58–0.85)	0	395	0.70 (0.54–0.89)	Never smoker	157	0.96 (0.66–1.41)
										Female	388	0.89 (0.70–1.12)	≥65	442	0.76 (0.61–0.95)	1	763	0.77 (0.65–0.90)	Current/previous smoker	996	0.72 (0.62–0.84)
2020-Herbst -N Engl J Med	IMpower110	NCT02409342	3	572	NSCLC	1	Atezolizumab vs. chemotherapy (high PD-L1 expression)	205	0.59 (0.40–0.89)	Male	143	0.57 (0.35–0.93)	<65	102	0.59 (0.34–1.04)	0	73	0.42 (0.20–0.92)	Never	24	1.83 (0.63–5.31)
										Female	62	0.69 (0.34–1.39)	65-74	80	0.63 (0.34–1.19)	1	132	0.69 (0.43–1.10)	Current	49	0.35 (0.14–0.88)
																			Previous	132	0.60 (0.36–1.00)
2021-Nishio-J Clin Oncol	IMpower132	NCT02657434	3	578	Nonsquamous	1	Atezolizumab+carboplatin/cisplatin+pemetrexed vs. carboplatin/cisplatin+pemetrexed	578	0.86 (0.71, 1.06)	Male	384	0.93 (0.73, 1.18)	<65	321	0.88 (0.67, 1.16)	0	240	0.76 (0.55, 1.04)	Never smoker	67	0.78 (0.42, 1.43)
										Female	194	0.76 (0.54, 1.09)	≥65	257	0.84 (0.63, 1.13)	1	336	0.99 (0.76, 1.27)	Current/former smoker	511	0.89 (0.72, 1.09)

Abbreviations: HR = hazard ratio; OS = overall survival; ABCP =atezolizumab plus bevacizumab plus carboplatin plus paclitaxel; ACP =atezolizumab plus carboplatin plus paclitaxel; BCP =bevacizumab plus carboplatin plus paclitaxel.

**Table 2:** Differences in overall survival associated with immunotherapy in sex by subgroups.

		Participants, no.		Pooled HR (95% CI) for ICI vs. controlled therapies		Test for difference
Variable	Studies, no.	Male	Female	Male	Female	*P* value
Overall	18	8881	4430	0.77 (0.72, 0.82)	0.77 (0.67, 0.87)	0.955
Line of therapy						
First	11	5460	2748	0.78 (0.72, 0.86)	0.74 (0.61, 0.89)	0.596
Subsequent	7	3421	1682	0.75 (0.69, 0.82)	0.80 (0.67, 0.96)	0.571
Intervention therapy						
ICI alone	11	5128	2717	0.74 (0.68, 0.80)	0.82 (0.73, 0.92)	0.13
ICI combined with non-ICI	7	3753	1713	0.83 (0.76, 0.90)	0.68 (0.51, 0.91)	0.201
Pathologic types						
Squamous	4	1855	408	0.76 (0.63, 0.93)	0.73 (0.46, 1.17)	0.886
Nonsquamous	5	2425	1605	0.80 (0.72, 0.89)	0.67 (0.51, 0.89)	0.252

Abbreviations: HR = hazard ratio; ICI = immune checkpoint inhibitor.

**Table 3:** Differences in overall survival associated with immunotherapy in age by subgroups.

		Participants, no.		Pooled HR (95% CI) for ICI vs. controlled therapies		Test for difference
Variable	Studies, no.	<65	≥65	<65	≥65	*P* value
Overall	18	7092	5715	0.74 (0.68, 0.81)	0.80 (0.75, 0.86)	0.17
Line of therapy						
First	11	4286	3561	0.74 (0.65, 0.85)	0.80 (0.73, 0.87)	0.351
Subsequent	7	2806	2154	0.73 (0.64, 0.83)	0.80 (0.69, 0.92)	0.367
Intervention therapy						
ICI alone	11	4268	3369	0.74 (0.66, 0.83)	0.78 (0.72, 0.85)	0.468
ICI combined with non-ICI	7	2824	2346	0.73 (0.63, 0.86)	0.83 (0.74, 0.94)	0.203
Pathologic types						
Squamous	4	1112	973	0.69 (0.53, 0.91)	0.82 (0.65, 1.05)	0.358
Nonsquamous	5	2203	1664	0.74 (0.62, 0.88)	0.77 (0.67, 0.87)	0.72

Abbreviations: HR = hazard ratio; ICI = immune checkpoint inhibitor.

**Table 4:** Differences in overall survival associated with immunotherapy in ECOG PS by subgroups.

		Participants, no.		Pooled HR (95% CI) for ICI vs. controlled therapies		Test for difference
Variable	Studies, no.	ECOG 0	ECOG ≥ 1	ECOG 0	ECOG ≥ 1	*P* value
Overall	18	4853	8414	0.77 (0.71, 0.84)	0.76 (0.70, 0.82)	0.765
Line of therapy						
First	11	3014	5165	0.76 (0.67, 0.87)	0.78 (0.71, 0.86)	0.768
Subsequent	7	1839	3249	0.79 (0.70, 0.89)	0.73 (0.63, 0.85)	0.432
Intervention therapy						
ICI alone	11	2723	5102	0.76 (0.69, 0.84)	0.76 (0.68, 0.85)	0.993
ICI combined with non-ICI	7	2130	3312	0.79 (0.67, 0.93)	0.78 (0.70, 0.88)	0.977
Pathologic types						
Squamous	4	711	1543	0.79 (0.58, 1.09)	0.73 (0.65, 0.89)	0.66
Nonsquamous	5	1662	2377	0.72 (0.61, 0.86)	0.78 (0.67, 0.90)	0.513

Abbreviations: HR = hazard ratio; ICI = immune checkpoint inhibitor.

**Table 5:** Differences in overall survival associated with immunotherapy in smoking status by subgroups.

		Participants, no.		Pooled HR (95% CI) for ICI vs. controlled therapies		Test for difference
Variable	Studies, no.	Smoker	Never smoker	Smoker	Never smoker	*P* value
Overall	15	8679	1439	0.77 (0.71, 0.83)	0.85 (0.70, 1.03)	0.335
Line of therapy						
First	10	5861	965	0.78 (0.71, 0.87)	0.80 (0.63, 1.01)	0.881
Subsequent	5	2818	474	0.74 (0.68, 0.82)	0.93 (0.60, 1.45)	0.334
Intervention therapy						
ICI alone	10	5743	1020	0.75 (0.69, 0.83)	0.99 (0.81, 1.21)	0.019
ICI combined with non-ICI	5	2936	419	0.79 (0.70, 0.90)	0.67 (0.48, 0.92)	0.032
Pathologic types						
Squamous	2	877	55	0.73 (0.50, 1.07)	0.85 (0.43, 1.68)	0.701
Nonsquamous	5	2767	482	0.76 (0.67, 0.86)	0.70 (0.50, 0.97)	0.626

Abbreviations: HR = hazard ratio; ICI= immune checkpoint inhibitor.
